# Supplementary material for: Fitness adaptations of Japanese encephalitis virus in pigs following vector-free serial passaging
Source: PLoS Pathog. 2024 Aug 26;20(8):e1012059. doi: 10.1371/journal.ppat.1012059 (PMC11379391; doi:10.1371/journal.ppat.1012059)
Supplement: S1 Text — (PDF) [file ppat.1012059.s001.pdf]

Supplementary Methods for “Fitness adaptations of Japanese encephalitis virus in pigs following vector-free serial passaging”

S1 Methods: Internal risk-benefit evaluation

The authors of this study, in collaboration with IVI’s biosafety unit, performed an internal risk-benefit evaluation to address the possibility that viruses with altered and potentially more dangerous biological characteristics could emerge during the serial DT experiments. The following factors were identified in favor of performing the present work: (1) As explained in the introduction, there is clear evidence of DT of JEV between pigs. Considering the long duration of shedding [1,2] and the high densities of animals in pig farming, it is a realistic scenario that JEV could undergo a similar evolutionary pressure in the field as modelled experimentally in the present study. In the field, this scenario would happen without any protection for the environment, the animal and human populations. Considering that in large parts of the world, the bird, pig and human populations are immunologically naïve, the emergence of a JEV with increased virulence and transmissibility would represent a catastrophic scenario. The present study was therefore seen as an important contribution to risk evaluation for epidemic preparedness against JEV and possibly other Flaviviruses. (2) The IVI containment facility operates at the highest possible BSL4 with respect to the protection of the environment. A virus escape from the laboratory building is considered as impossible or an extremely low risk. (3) All work inside this containment was performed using PPE, fulfilling at least BSL3 requirements. All persons involved in the present work were protected by full-body, HEPA-filtered air-supplied suits. As an additional safety, all personnel involved in the experimentation were vaccinated against JEV. Considering these points, we concluded that on one side, the risk of virus escape was extremely low, and on the other side, the gain in important knowledge required for pandemic preparedness can be crucial to evaluate the risks caused by JEV and develop mitigation strategies against this risk.

S2 Methods: Clinical scores

The checklist for clinical scores of JEV-infected pigs was as previously described [1].

Reference: Ricklin et al., 2016, Nat Commun 7: 10832; Ricklin et al. 2016, Vet Res 47: 34

Instructions:

Monitoring: After infection and on all days with clinical symptoms, examination of the pigs is performed at least once a day every day by a veterinarian (twice a day if criteria for discontinuation may be reached before the next day). When symptoms occur, monitoring is performed by the same person whenever possible to ensure unbiased clinical assessment. Focus is placed on neurological symptoms in particular reflected by parameters 1, 2, 4.

Criteria for discontinuation of the experiment: A pig will be euthanized immediately as soon as it reaches a severity degree of 3, *i.e.*
-> a total clinical score of 16 or higher for all parameters together
-> or a score of 3 for at least one of the parameters #1, 2 or 4

Parameter	Criteria	Score
1. Liveliness	• Attentive (curious, stands up immediately)	0
	• Slightly reduced (stands up hesitantly, but without help)	1
	• Tired, gets up only when forced to, lies down again	2
	• Somnolent, won't stand up for more than 24 h	3
2. Body tension	• Relaxed, straight back	0
	• Stiffness and bent back while standing up, afterwards normal	1
	• Bent back and stiff walking remains	2
	• Prolonged cramps of neurological origin, unable to walk	3
3. Body shape	• Full stomach, "round" body	0
	• Empty stomach	1
	• Empty stomach, thinned body muscles	2
	• Emaciated, backbone and ribs clearly visible	3
4. Walking, Neurological symptoms	• Well coordinated movements	0
	• Hesitant walking, crossed-over legs are corrected slowly	1
	• Distinct ataxia/hind lameness, able to walk	2
	• Massive lameness, unable to walk for more than 24 h	3
5. Appetite	• Greedy, hungry	0
	• Eats slowly when fed	1
	• Doesn't eat when fed, but sniffs food	2
	• Doesn't eat at all, shows no interest for food	3
6. Defecation	• Soft faeces, normal amount	0
	• Reduced amount of faeces, dry	1
	• Only small amount of dry, fibrin-covered faeces, or diarrhoea	2
	• No faeces, mucus in rectum, or watery and bloody diarrhoea	3
7. Leftovers in feeding trough	• Trough empty, clean	0
	• Trough almost empty, almost no leftovers	1
	• Food only partially eaten	2
	• Trough still full, nothing eaten	3

S3 Methods: Cell culture media

C6/36 cells were cultured in in minimal essential medium (MEM, Thermo Fisher Scientific) supplemented with non-essential amino acid solution (NEAA, Thermo Fisher), 100mM sodium pyruvate (Thermo Fisher) and 10% fetal bovine serum (FBS, Thermo Fisher). PEDSV.15 cells were cultured at 37°C and 5% CO_2 in Dulbecco's modified eagle medium containing GlutaMAX (DMEM, Thermo Fisher), NEAA, sodium pyruvate, 7% horse serum (Biowest) and 2% porcine serum (Sigma-Aldrich). During infection, the media contained only 2% horse serum. Vero cells were cultured at 37°C and 5% CO_2 in DMEM containing GlutaMAX and 10% FBS. Monocytes were then differentiated into macrophages by incubation for four days at 39°C and 5% CO_2 in DMEM/10% FBS and 20 U/ml porcine M-CSF (produced in-house). [3] During infection the serum concentration of all cell cultures was reduced to 2%.

S4 Methods: Construction of JEV RNA for quantitative RT-PCR

For PCR amplification from JEV cDNA, Phusion Hot Start II DNA Polymerase (ThermoFisher Scientific), dNTP mix (ThermoFisher Scientific), the forward primer 5' - CTC

ATC GGA ACG CGA TCC AG – 3’ and the reverse primer 5’- TAG ACG AGG CTT GGG CCG TGG G – 3’ were used. The PCR followed 32 cycles, with an annealing temperature of 57°C. For the adenylation of the PCR fragments, the reaction was incubated for 10 minutes at 72°C with a Taq polymerase (Thermo Fisher) prior to pCR4-TOPO cloning. The TOPO cloning was performed using the TOPO TA cloning Kit for Sequencing (Thermo Fisher). Briefly, after overnight incubation of the purified PCR product (4µl) with 1µl of pCR™4-TOPO TA vector, 2µl of the reaction was used to transform electrocompetent TOP10 *E. coli* cells. After kanamycin selection on LB-agar plates, single colonies were used to inoculate liquid LB-kanamycin cultures for the amplification and subsequent isolation of plasmid DNA. To select positive clones, the plasmids were digested with the restriction enzyme *EcoR*I (ThermoFisher), and the resulting DNA fragments were separated by gel electrophoresis. To screen for the correct orientation of the insert within the plasmid, miniprep DNA preparations were digested with the restriction enzymes *Tth111*I and *Not*I (Thermo Fisher) and *Sfi*I/*Not*I (Thermo Fisher). The final construct was verified by Sanger sequencing using pCR4-TOPO-specific M13_F and M13_R oligonucleotides. The RNA was synthesized using the T7 RNA Polymerase (MEGAscript T7 Transcription Kit, Ambion) with a *Bcu*I linearized, phenol/chloroform-extracted plasmid template. The RNA was further purified by two rounds of RNA extraction using the NucleoSpin RNA Kit (Macherey Nagel). The purity and integrity of the RNA was analyzed by gel electrophoresis. The concentration of the RNA was determined using a Nanodrop 2000C Spectrometer (Thermo Fisher).

S5 Methods: Transcriptomics

To isolate blood leukocytes, EDTA blood was treated with a red blood cell lysis buffer (1.5M NH_4Cl, 100mM $NaHCO_3$, 10mM EDTA in H_2O) at 37°C for 2.5 minutes to obtain complete erythrocyte lysis. The remaining cells were washed three times with cold Dulbecco′s Phosphate Buffered Saline (PBS, Thermo Fisher) before lysing the pellet in Trizol (Thermo Fisher). After the addition of a 49:1 mixture of chloroform (Sigma) and isoamyl alcohol (Sigma), the aqueous phase was transferred, and an equal volume of 75% ethanol (Merck) was added. Then, RNA was finally extracted by loading the mixture on the column of the Nucleospin RNA extraction Kit following the manufacturer’s instructions. The RNA was then sent for Bulk RNA barcoding and sequencing (BRB-seq) at the sequencing center of the University of Bern. A quality control by fragment analysis (5200 Fragment Analyzer CE instrument, Agilent) was performed prior to the sequencing with the Illumina NovaSeq6000 sequencer (Illumina). Reads were mapped to the pig genome (*Sus scrofa* 11.1, Ensembl release) using Tophat v.2.0.11 [4–7]. The number of reads overlapping with each gene was evaluated with Htseq-count v.0.6.1 [8,9]. The Bioconductor package DESeq2 v1.38.3 [10] was used to test for differential gene expression between the experimental groups. Gene set enrichment analysis (GSEA) was performed following ranking of genes based on differential gene expression using the “stat” value [11,12]. Calculations of normalized enrichment scores and false discovery rates (FDR) was performed using online tools available on https://www.gsea-msigdb.org [13]. As gene sets we employed the blood transcriptional modules (BTM) defined by Li *et al*. [14] for humans and the modified version for pigs as previously described [15]. Figures were created in R 4.3.0 using ggplot2 Bioconductor package.

S6 Methods: Histopathology

Half of the brain (including one complete cerebral and cerebellar hemisphere) and cervical spinal cord, tonsil and mandibular lymph node were fixed in 4% buffered formalin for four weeks. For histopathological examination cross sections of collected tissues were performed, including representative areas of the central nervous system (CNS): olfactory bulb, frontal lobe, basal nuclei, hippocampus, cerebral cortex, thalamus, midbrain, pons, cerebellum and cervical spinal cord. The tissue sections were embedded in paraffin, cut at 4 µm of thickness and stained with hematoxylin and eosin (HE). Lesions in the CNS were semi-quantitatively scored from 0 to 3, as previously described [16], with minor modifications: 0 = no lesions, 1 = mild lesions, 2 = moderate lesions, and 3 = severe lesions.

Grade 1 corresponds to few and thin perivascular cuffs and/or few glial nodules. Grade 2 is characterized by prominent perivascular cuffs and multiple dense multifocal glial nodules with neuronal degeneration and neuronophagia. Grade 3 corresponds to extended lesions with prominent perivascular cuffs and numerous glial nodules that may coalesce, and frequent evidence of neuronophagia. All scoring was done as blind trial. A sum of the scores per pig was calculated by adding all the scored areas as previously described. [16]

S7 Methods: Serum neutralization assay

Firstly, focus forming units of JEV Laos stocks were determined by tenfold serially diluting the virus stock in MEM starting at 1:100 dilution in quadruplicates. 200µl of this viral dilution was added to confluent Vero cells, seeded in a 24-well plate. The inoculum was incubated for 1.5 hours at 37°C and 5% CO_2. Thereafter, the cells were washed with pre-warmed D-PBS and incubated with 1ml of medium supplemented with 1% methylcellulose (Sigma Aldrich) at 37°C and 5% CO_2 for 72 hours. Then, the overlay medium was aspirated, cells were washed once with pre-warmed PBS and fixed with 4% buffered formalin solution (Polyscience Inc). The virus infected cells were stained by an immuno-peroxidase staining, described in the S9 Method. The foci were counted and multiplied by the dilution to calculate the Focus forming units per milliliter (FFU/ml).

For the neutralization test, all sera samples were treated at 56°C for 20 minutes to inactivate the complement. Then, sera were serially twofold diluted in media, starting with a 1/10 dilution. Thereafter, 200 FFU/ well of JEV Laos were added and the plates were incubated for 1h at 37°C with 5%CO_2. Each mix containing 100 FFU of JEV Laos was then pipetted to wells with confluent Vero cells and incubated for 48h at 37°C with 5% CO_2. After removal of the supernatant, the cells were washed once with PBS, before fixation with 4% buffered formalin solution. This was followed by E protein immune-peroxidase staining, as described in S9 Methods. Finally, the plates were photographed using the Immunospot® machine (Cellular Technology LTD), and the area of E protein staining was quantified for each well using Image J software. Wells with naïve sera were used as controls as negative controls, and wells were 100 FFU of JEV were added in absence of immune serum were included as positive controls. The serum dilution that allowed a reduction of at least 50% E protein staining was used as cut off. The 50% neutralizing dose (ND_{50}) was calculated using the Spearman-Kaerber formula [17].

S8 Methods: Viral titration

For the titration of JEV from cell culture supernatants, the cells were seeded in a 96-well plate. Once cell confluency was reached, the medium was replaced. Then, virus samples were ten-fold serially diluted, starting at 1:10 dilution in quadruplicates and incubated on the cells for three days. The cells were washed with PBS and fixed with 4% formalin. Finally, virus-infected cells were labelled by an immunoperoxidase-staining as described in S9 Methods. Wells containing stained JEV-infected cells were counted as positive, while wells with unstained cells were counted as negative. The number of positive and negative wells were used to determine the TCID50/ml using the Reed-Muench formula [18,19]

S9 Methods: Immunoperoxidase staining for JEV infected cells

The virus-infected cells were visualized by immunoperoxidase staining using the mouse anti-Flavivirus E protein monoclonal antibody (ATCC HB 112, D1-4G2-4-15) diluted in 0.3% saponin (PanReac AppliChem) solution in PBS for 30 min at 37°C. After 3 washes with wash buffer (0.14M NaCl, 1.47mM KH_2PO_4, 7.8mM Na_2HP_4, 2.68mM KCl and 0.05% v/v Tween 20), the rabbit anti-mouse HRP (Agilent Technology) diluted in 0.3% saponin solution in PBS was added on the cells and incubated for another 30 min at 37°C, then washed 3 times, as before. Then infected cells were visualized after 30 min of incubation in AEC solution in the dark. The AEC solution contains 50mM Na-Acetate with 5% v/v 3-Amino-9-ethylcarbazol dissolved in N,N-dimethylformamide (all Sigma) and 5% v/v H_2O_2.

S10 Methods: Virus isolation from sera

To enhance efficiency of virus isolation from sera, the samples were diluted 1/20 in DMEM and mixed with immune sera (0.1 % v/v) from vaccinated pigs that was previously demonstrated to strongly enhance JEV infection. [20] After 1 hour at 37°C the sera mix was added to monocyte derived macrophages (MDM) for another 1.5 h. Then, cells were washed twice with pre-warmed PBS and incubated with infection media for 72h at 37°C, 5% CO_2. This resulted in an effective JEV replication in MDM, allowing us to rescue the virus from all serum of viremic pigs. The rescued viruses were titrated like described in S8 Methods for the determination of the $TCID_{50}$/ml.

S11 Methods: Virus sequencing

For the viral genome sequencing, RNA was extracted from serum or swab samples using the TRIzol™ (Thermo Fisher). After the addition of a 49:1 mixture of Chloroform (Sigma) and isoamyl alcohol (Sigma), the aqueous phase was transferred, and an equal volume of 75% ethanol (Merck) was added. The mixture was then loaded onto the RNA column of the NucleoSpin RNA extraction Kit (Macherey Nagel). The RNA was extracted following the guidelines of the kit.

The quantity and quality of the purified total RNA was assessed using the Qubit 4.0 fluorometer with the Qubit RNA BR & HS Assay Kits (Thermo Fisher), and an Advanced Analytical Fragment Analyzer System using a Fragment Analyzer RNA Kit (Agilent). Input RNA samples varying between 0.5ng-1000ng were first depleted of ribosomal RNA and globin mRNA using RiboCop for HMR + Globin Depletion Kit following the Lexogen user guide (144UG288V0101). Thereafter, cDNA libraries were generated using a CORALL Total RNA-Seq V2 library Prep.kit with UDIs 12nt set A1-A4 (Lexogen) according to the protocol for short insert sizes and with 11-19 PCR cycles (Lexogen user guide 171UG394V0100). The resulting cDNA libraries were evaluated using a Qubit 4.0 fluorometer with the Qubit dsDNA HS Assay Kit (Thermo Fisher) and an Agilent Fragment Analyzer (Agilent) with a HS NGS Fragment Kit (Agilent), respectively. In a pilot experiment pooled libraries were sequenced 50 bp paired end using an Illumina NovaSeq 6000 SP Reagent Kit v1.5 (100 cycles; Illumina, 200228401) on an Illumina NovaSeq 6000 instrument. For the main experiment and majority of samples, the cDNA libraries were equimolar-pooled and sequenced 100 bp paired-end using a shared Illumina NovaSeq 6000 S4 Reagent Kit v1.5 (200 cycles; Illumina) on an Illumina NovaSeq 6000 instrument. The quality of the sequencing run was assessed using Illumina Sequencing Analysis Viewer (Illumina version 2.4.7) and all base call files were demultiplexed and converted into FASTQ files using Illumina bcl2fastq conversion software v2.20.

Supplementary References

1. Ricklin ME, García-Nicolás O, Brechbühl D, Python S, Zumkehr B, Nougairede A, et al. Vector-free transmission and persistence of Japanese encephalitis virus in pigs. Nat Commun. 2016;7: 10832. doi:10.1038/ncomms10832

2. Lyons AC, Huang Y-JS, Park SL, Ayers VB, Hettenbach SM, Higgs S, et al. Shedding of Japanese Encephalitis Virus in Oral Fluid of Infected Swine. Vector-Borne and Zoonotic Diseases. 2018;18: 469–474. doi:10.1089/vbz.2018.2283

3. Sautter CA, Trus I, Nauwynck H, Summerfield A. No Evidence for a Role for Antibodies during Vaccination-Induced Enhancement of Porcine Reproductive and Respiratory Syndrome. Viruses. 2019;11: 829. doi:10.3390/v11090829

4. Kim D, Pertea G, Trapnell C, Pimentel H, Kelley R, Salzberg SL. TopHat2: accurate alignment of transcriptomes in the presence of insertions, deletions and gene fusions. Genome Biol. 2013;14: R36. doi:10.1186/gb-2013-14-4-r36

5. Kim D, Salzberg SL. TopHat-Fusion: an algorithm for discovery of novel fusion transcripts. Genome Biol. 2011;12: R72. doi:10.1186/gb-2011-12-8-r72

6. Trapnell C, Pachter L, Salzberg SL. TopHat: discovering splice junctions with RNA-Seq. Bioinformatics. 2009;25: 1105–1111. doi:10.1093/bioinformatics/btp120

7. Langmead B, Trapnell C, Pop M, Salzberg SL. Ultrafast and memory-efficient alignment of short DNA sequences to the human genome. Genome Biol. 2009;10: R25. doi:10.1186/gb-2009-10-3-r25

8. Putri GH, Anders S, Pyl PT, Pimanda JE, Zanini F. Analysing high-throughput sequencing data in Python with HTSeq 2.0. Boeva V, editor. Bioinformatics. 2022;38: 2943–2945. doi:10.1093/bioinformatics/btac166

9. Anders S, Pyl PT, Huber W. HTSeq—a Python framework to work with high-throughput sequencing data. Bioinformatics. 2015;31: 166–169. doi:10.1093/bioinformatics/btu638

10. Love MI, Huber W, Anders S. Moderated estimation of fold change and dispersion for RNA-seq data with DESeq2. Genome Biol. 2014;15: 550. doi:10.1186/s13059-014-0550-8

11. Boccard J, Schvartz D, Codesido S, Hanafi M, Gagnebin Y, Ponte B, et al. Gaining Insights Into Metabolic Networks Using Chemometrics and Bioinformatics: Chronic Kidney Disease as a Clinical Model. Front Mol Biosci. 2021;8: 682559. doi:10.3389/fmolb.2021.682559

12. Subramanian A, Tamayo P, Mootha VK, Mukherjee S, Ebert BL, Gillette MA, et al. Gene set enrichment analysis: A knowledge-based approach for interpreting genome-wide expression profiles. Proc Natl Acad Sci USA. 2005;102: 15545–15550. doi:10.1073/pnas.0506580102

13. Benjamini Y, Hochberg Y. Controlling the False Discovery Rate: A Practical and Powerful Approach to Multiple Testing. Journal of the Royal Statistical Society: Series B (Methodological). 1995;57: 289–300. doi:10.1111/j.2517-6161.1995.tb02031.x

14. Li S, Rouphael N, Duraisingham S, Romero-Steiner S, Presnell S, Davis C, et al. Molecular signatures of antibody responses derived from a systems biology study of five human vaccines. Nat Immunol. 2014;15: 195–204. doi:10.1038/ni.2789

15. Matthijs AMF, Auray G, Jakob V, García-Nicolás O, Braun RO, Keller I, et al. Systems Immunology Characterization of Novel Vaccine Formulations for Mycoplasma hyopneumoniae Bacterins. Front Immunol. 2019;10: 1087. doi:10.3389/fimmu.2019.01087

16. Ricklin ME, Garcìa-Nicolàs O, Brechbühl D, Python S, Zumkehr B, Posthaus H, et al. Japanese encephalitis virus tropism in experimentally infected pigs. Vet Res. 2016;47: 34. doi:10.1186/s13567-016-0319-z

17. Ramakrishnan MA. Determination of 50% endpoint titer using a simple formula. WJV. 2016;5: 85. doi:10.5501/wjv.v5.i2.85

18. Reed LJ, Muench H. A SIMPLE METHOD OF ESTIMATING FIFTY PER CENT ENDPOINTS12. American Journal of Epidemiology. 1938;27: 493–497. doi:10.1093/oxfordjournals.aje.a118408

19. Lei C, Yang J, Hu J, Sun X. On the Calculation of TCID50 for Quantitation of Virus Infectivity. Virol Sin. 2021;36: 141–144. doi:10.1007/s12250-020-00230-5

20. García-Nicolás O, Ricklin M, Liniger M, Vielle N, Python S, Souque P, et al. A Japanese Encephalitis Virus Vaccine Inducing Antibodies Strongly Enhancing In Vitro Infection Is Protective in Pigs. Viruses. 2017;9: 124. doi:10.3390/v9050124
