# Supplementary material for: Fitness adaptations of Japanese encephalitis virus in pigs following vector-free serial passaging
Source: PLoS Pathog. 2024 Aug 26;20(8):e1012059. doi: 10.1371/journal.ppat.1012059 (PMC11379391; doi:10.1371/journal.ppat.1012059)
Supplement: S1 Fig — (PDF) [file ppat.1012059.s002.pdf]

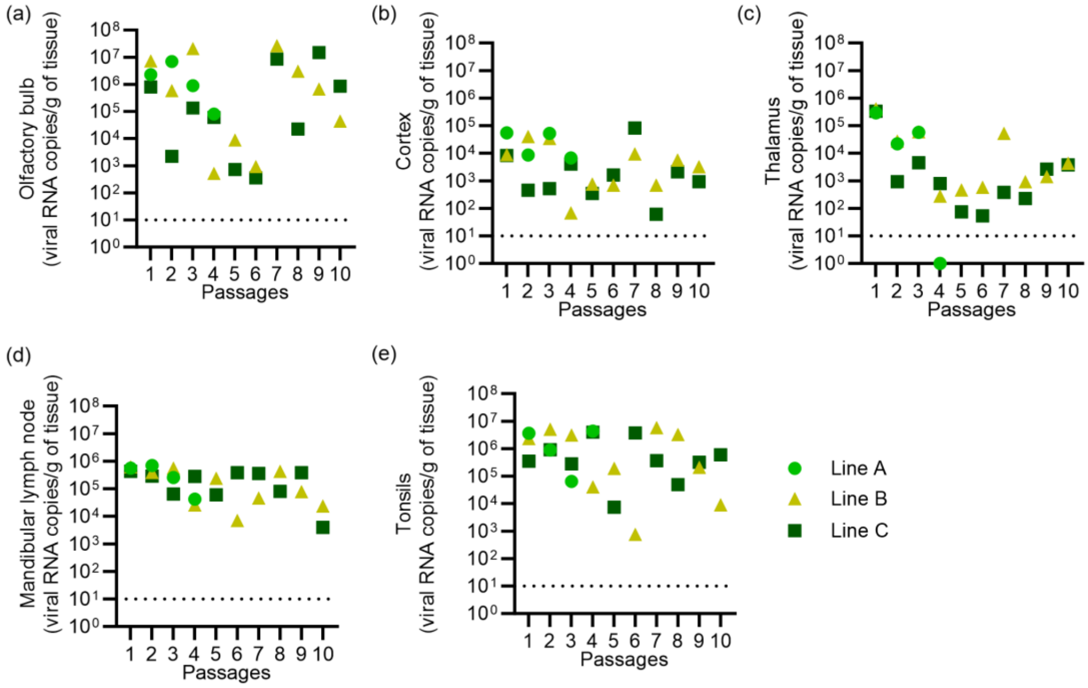

S1 Fig: Tissue distribution of JEV RNA during passaging. In (a)-(e) RNA loads in tissues of the olfactory bulb, the cortex, the thalamus, the mandibular lymph nodes and the tonsils, respectively, are shown. Due to the loss of line A during passaging, only 2 datapoints were left. Therefore, no statistical analysis was performed with this data.
