# Supplementary material for: Fitness adaptations of Japanese encephalitis virus in pigs following vector-free serial passaging
Source: PLoS Pathog. 2024 Aug 26;20(8):e1012059. doi: 10.1371/journal.ppat.1012059 (PMC11379391; doi:10.1371/journal.ppat.1012059)
Supplement: S2 Fig — (PDF) [file ppat.1012059.s003.pdf]

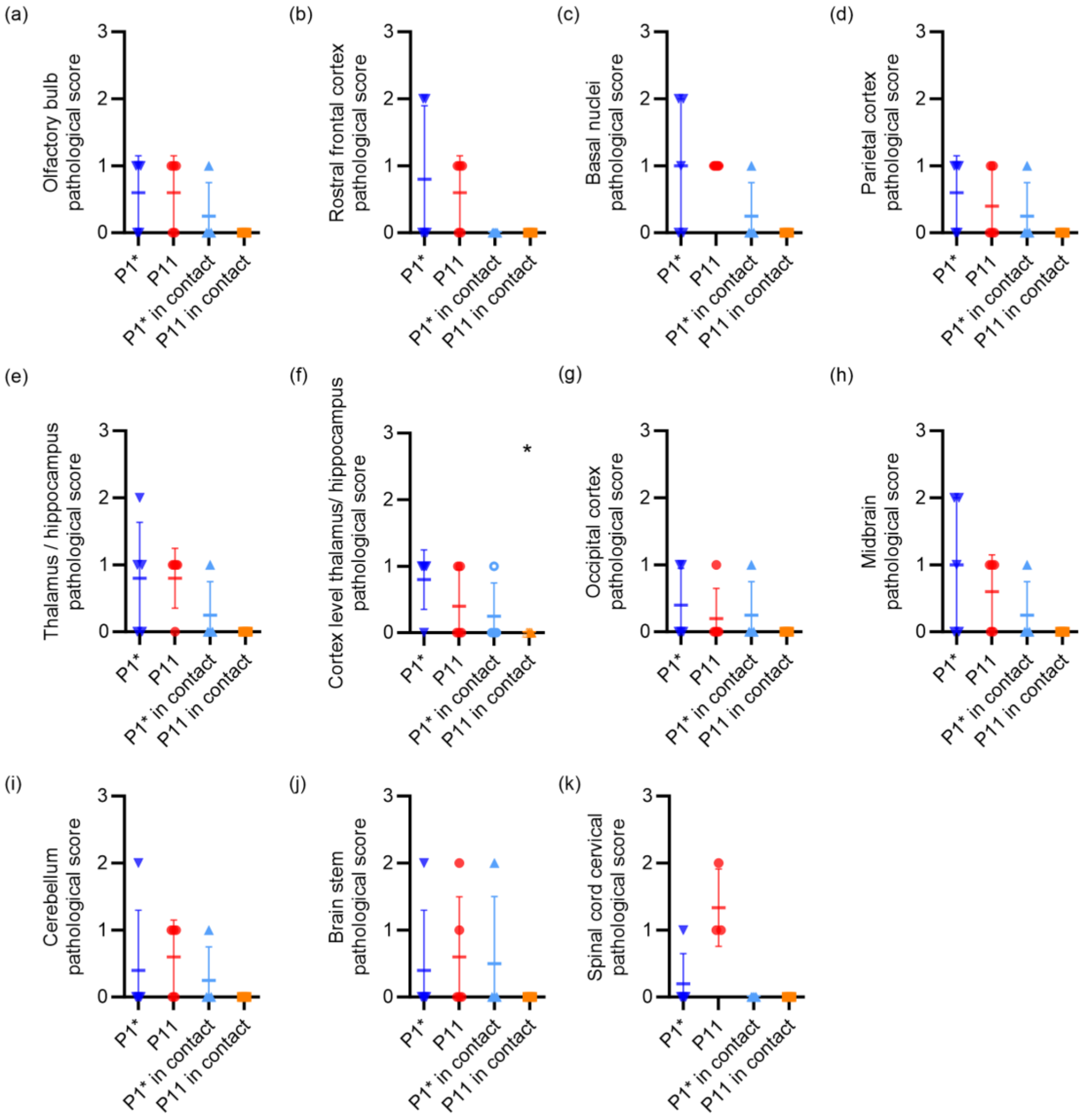

S2 Fig: Histopathological analyses of CNS from JEV infected pigs. Formalin-fixed sections of the olfactory bulb (a), the rostral frontal cortex (b), the basal nuclei (c), the parietal cortex (d), the thalamus/ hippocampus (e), the cortex level of thalamus/ hippocampus (f), the occipital cortex (g), the midbrain (h), the cerebellum (i), the brain stem (j) and the spinal cord cervical (k) were embedded in paraffin, cut at 4 µm and HE-stained. Lesions were semi-quantitatively scored from 0 to 3 (0 = no lesions, 1 = mild, 2 = lesions, and 3 = severe lesions). For the cervical spinal cord, the P10 group is missing two samples. Statistical analyses were performed using Mann-Whitney U test comparing each condition to P1*. The significance cut-off was set at $p < 0.05$.
