# Supplementary material for: Fitness adaptations of Japanese encephalitis virus in pigs following vector-free serial passaging
Source: PLoS Pathog. 2024 Aug 26;20(8):e1012059. doi: 10.1371/journal.ppat.1012059 (PMC11379391; doi:10.1371/journal.ppat.1012059)
Supplement: S3 Fig — (PDF) [file ppat.1012059.s004.pdf]

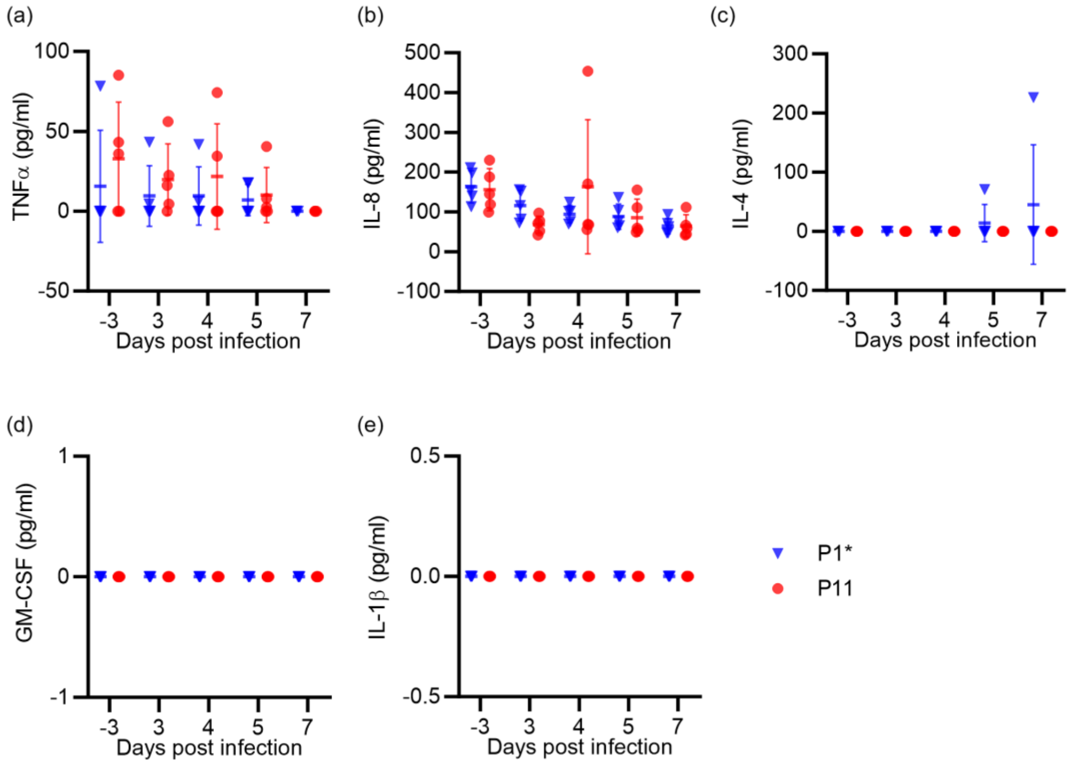


**S3 Fig: Unmodulated or undetectable cytokines in the serum of JEV-infected pigs.** Data for TNF (a), IL-8 (b), IL-4 (c) GM-CSF (d) and IL-1β (e) are shown. Statistical analysis was performed with Mann-Whitney U test comparing on one hand the different timepoints within the same group to d-3 and on the other hand the same timepoints between the group with each other. No significant increases in cytokine production ($p<0.05$) were found.

## Supplementary Figure 4

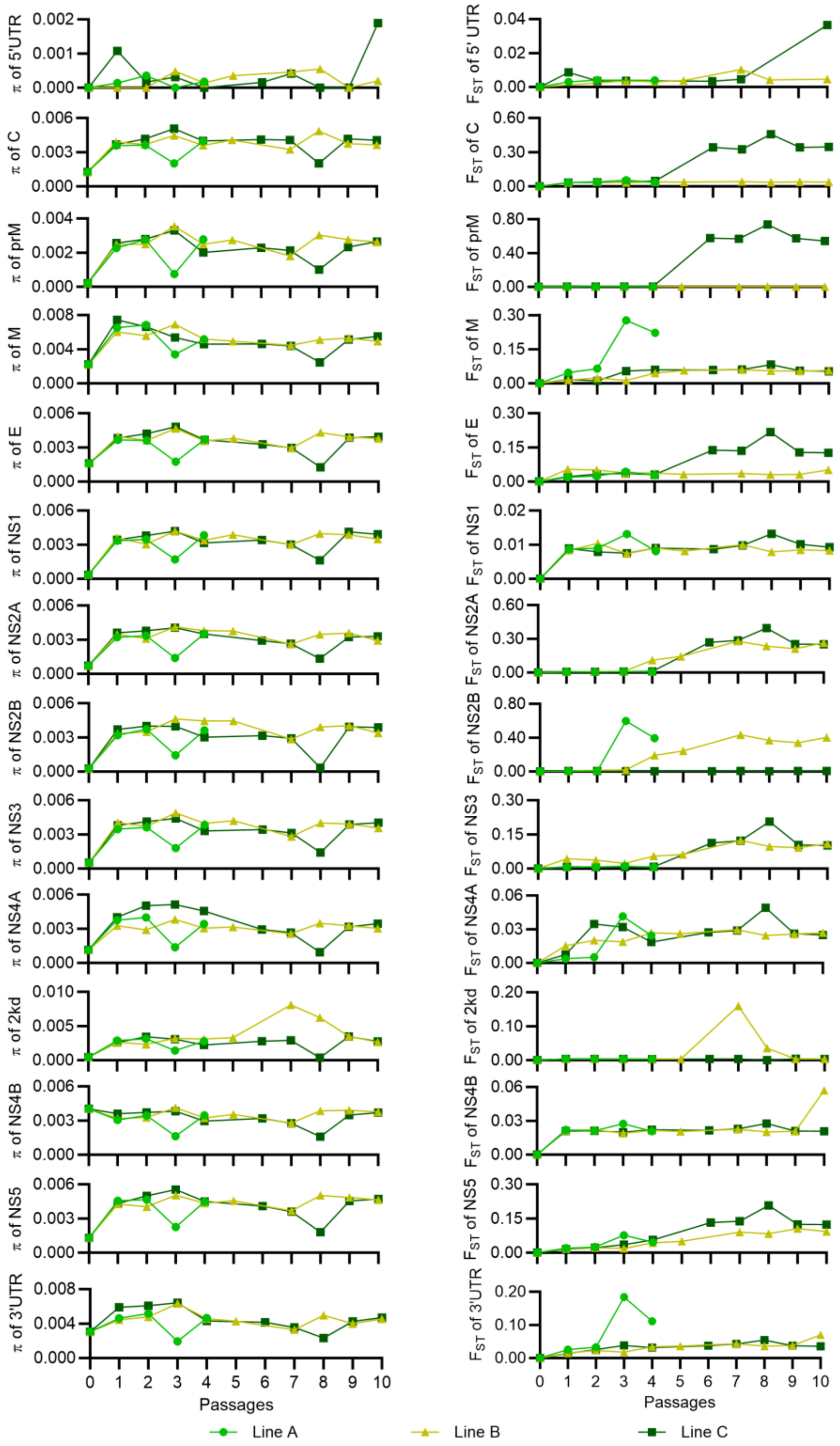


**S4 Fig: Nucleotide diversity π and fixation index $F_{ST}$ for individual viral genes and UTRs.** Viral RNA of d3 post-infection was analyzed by next generation sequencing. For each viral gene and the UTRs, the nucleotide diversity π (plots on the left), and the pairwise genetic differentiation between viral populations in P0 and each passage is shown as fixation index $F_{ST}$ (plots on the right).
