# Supplementary material for: Fitness adaptations of Japanese encephalitis virus in pigs following vector-free serial passaging
Source: PLoS Pathog. 2024 Aug 26;20(8):e1012059. doi: 10.1371/journal.ppat.1012059 (PMC11379391; doi:10.1371/journal.ppat.1012059)
Supplement: S4 Fig — (PDF) [file ppat.1012059.s005.pdf]

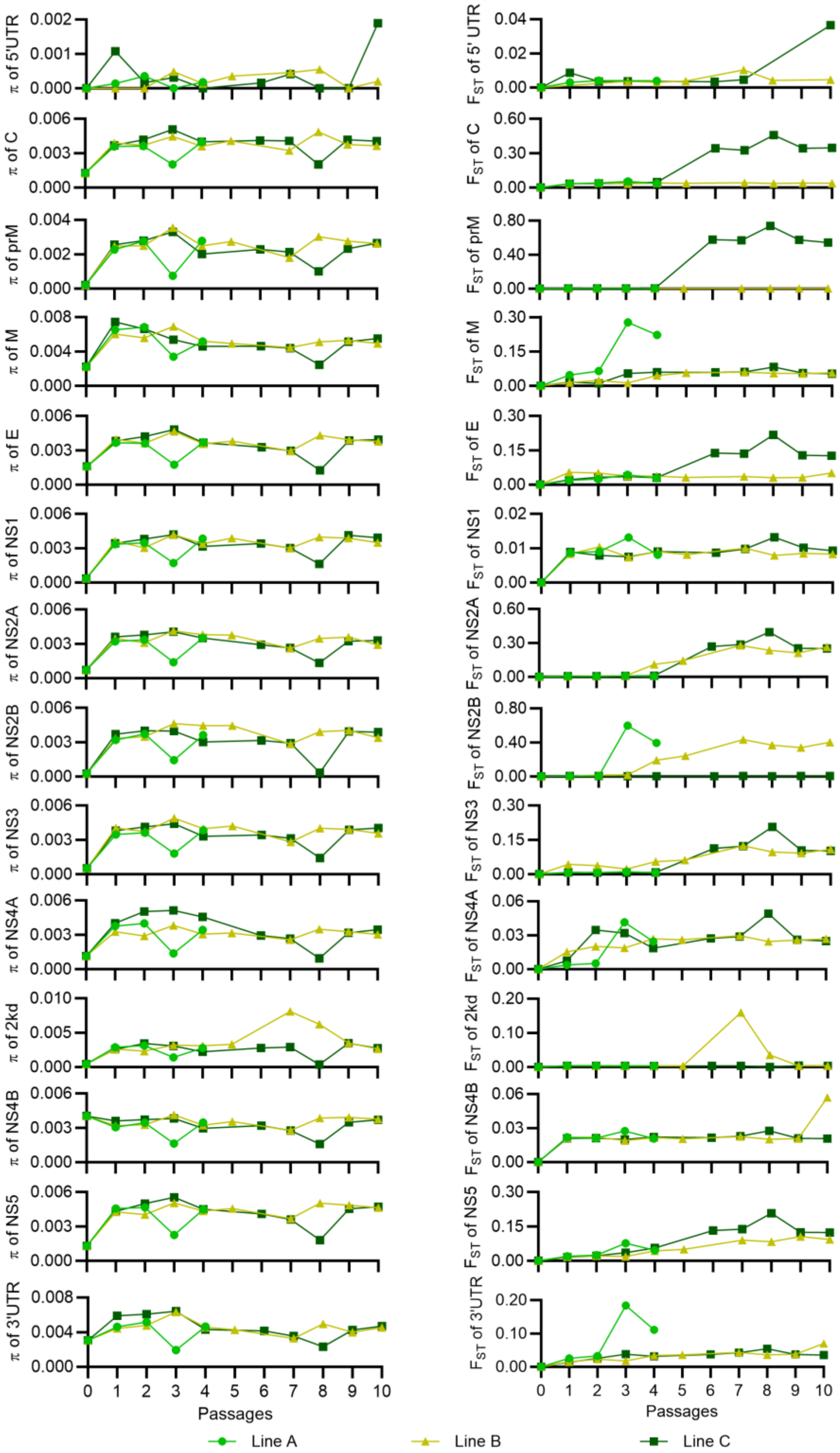

S4 Fig: Nucleotide diversity π and fixation index F_{ST} for individual viral genes and UTRs. Viral RNA of d3 post-infection was analyzed by next generation sequencing. For each viral gene and the UTRs, the nucleotide diversity π (plots on the left), and the pairwise genetic differentiation between viral populations in P0 and each passage is shown as fixation index F_{ST} (plots on the right).
