# Supplementary material for: Fitness adaptations of Japanese encephalitis virus in pigs following vector-free serial passaging
Source: PLoS Pathog. 2024 Aug 26;20(8):e1012059. doi: 10.1371/journal.ppat.1012059 (PMC11379391; doi:10.1371/journal.ppat.1012059)
Supplement: Attachment — Submitted filename: ReviewComments.docx [file ppat.1012059.s014.docx]

This study assessed the impacts of direct transmission of Japanese encephalitis virus (JEV) between pigs on virus adaptation to this mode of transmission. The authors demonstrated fitness gains for direct transmission. I think the study is of sufficient quality, originality, and interest for publication in PLoS Pathogens. I have no major comments with respect to the experimental design and the manuscript is well written but note that I do not have the skills and experience to comment comprehensively on the transcriptomics and genome analyses parts of the study. Please find comments detailed below. My main comments are with respect to the statistical analyses.

**Introduction**

Line 61: give reference for mortality rate.

Line 63: vertebrate ‘species’. I would not refer to transmission as dual-host – it is host-vector, also drop ‘alternating’ as you say cycling – same thing.

Line 67: must be adapted to both host and vector (refer to mosquitoes as vectors, not hosts).

Line 69: ‘and produce an immune response’.

Line 76: just ‘direct transmission’.

Line 77: omit ‘worrisome’.

Lines 80 to 82: Not necessarily true – this depends on pig population turnover and seasonal dynamics of JEV. In some regions if there is only seasonal transmission season, this could result in a build-up of susceptible animals between outbreaks. I would say it is less likely in many endemic areas because pig farming is not intensive.

**Methods**

Statistical analysis: it would be good to elaborate on the statistics in more detail. Clearly outline each hypothesis/ question each test is addressing. For example, ‘the analyses related to the *in vivo* passaging…’ separate this out into each analysis – what were the dependent variables? From reading the results it looks like analysis was done separately for individual time points for the dependent variables in Fig. 1, Fig. 2 and Fig. 3. Or, for the data in Fig. 1 did you only compare values at day 3-4? Ideally, you should treat time as a continuous variable and account for repeated measures in your statistical analyses. How were the curves generated in Fig. 3?

For *in vitro* growth curve analysis for example, you could follow the approach in Wang and Bushman (2006) A statistical method for comparing viral growth curves. Journal of Virological Methods. 135, 118-123. However, note this approach only examines the exponential phase of growth curves.

**Results**

Linked with the comments on statistics, it would be good to focus in the description of the results on effect sizes and present more quantitative findings. For example, lines 269 – 270 – what was the observed difference in growth rate? Line 291: significantly increased body temperature – but what was the difference in body temperature?
