# Supplementary material for: Fitness adaptations of Japanese encephalitis virus in pigs following vector-free serial passaging
Source: PLoS Pathog. 2024 Aug 26;20(8):e1012059. doi: 10.1371/journal.ppat.1012059 (PMC11379391; doi:10.1371/journal.ppat.1012059)
Supplement: Attachment — Submitted filename: Marti_reviewer_comments_answers.docx [file ppat.1012059.s015.docx]

**Point-by-point reply to the reviewer’s critique and requests for manuscript “Fitness adaptations of Japanese encephalitis virus in pigs following vector-free serial passaging”**

**Part I - Summary**
 **Reviewer #1:** This is the manuscript from the group that discovered and published the vector-free JEV transmission in pigs. The authors build on their expertise and further extended studies to serial passaging. The experimental design partially reproduces the possible non-vector transmissions on JEV-affected swine herds in endemic countries outside the mosquito season and models potential transmission events after introduction to naïve herds. The authors demonstrated that after serial passaging in pigs, JEV induced enchased clinical signs in some experimental conditions and altered immune responses. Using NGS, the emergence of new JEV variants was also identified.
The authors applied a well-established toolbox for JEV research and NGS, providing interesting results. The study was conducted in the native amplifying host—pigs. Counting that JEV is a BSL3 pathogen, this is a unique study because only a small number of research groups can conduct studies with BSL3 pathogens at such level in the native hosts, particularly large agricultural animals.

**Reviewer #2:** This research team previously demonstrated that the Japanese encephalitis virus (JEV) can undergo vector-free transmission among experimental pigs. This manuscript explores the changes in viral phenotype and molecular level after 10 passages of JEV transmission among pigs through direct (vector-free) transmissions. While the innovative research objective is appealing, the results lack persuasiveness, possibly due to the influence of pig experiments and specimen analysis methods, as well as insufficient in-depth discussion of the research findings.

**Reviewer #3:** This study assessed the impacts of direct transmission of Japanese encephalitis virus (JEV) between pigs on virus adaptation to this mode of transmission. The authors demonstrated fitness gains for direct transmission. I think the study is of sufficient quality, originality, and interest for publication in PLoS Pathogens. I have no major comments with respect to the experimental design and the manuscript is well written but note that I do not have the skills and experience to comment comprehensively on the transcriptomics and genome analyses parts of the study. Please find comments detailed below. My main comments are with respect to the statistical analyses.

**Authors:** We are very grateful to all reviewers for taking the time to read our manuscript and provide us with feedback that has helped us improve the manuscript

**Part II – Major Issues: Key Experiments Required for Acceptance**

**Reviewer #1:**

(No Response)

**Reviewer #2:**

General comments:
1. When exploring the impact of transmission between a single vector or vertebrate host, or between dual hosts, on the evolution of arboviruses, three sets of experiments should be conducted: virus infection only in the vector, virus infection only in the vertebrate host, and circulation infection between the dual hosts. In this study, the experiment only investigated JEV infection among pigs, without conducting the other two sets of experiments (at least the circulation infection between the vector and pigs should be conducted, as the probability of vertical transmission of JEV between vectors is very low). Therefore, it is difficult to infer that the observed changes in JEV phenotype and genotype are solely due to direct transmission of JEV among pigs.

**Authors:** We agree that a line of passaging using pigs and mosquitos in an alternating fashion would be a good control to be certain that the adaptations observed were really caused by the single host cycling only. Nevertheless, we were unable to include this line of passaging for practical reasons. The titers of JEV in the serum of pigs are too low to ensure artificial infection of mosquitos by blood feeding, and would therefore have required a problematic cell culture step to amplify the virus titers. Furthermore, we do not have the possibilities to do mosquito-pig transmission experiments at our institute. We have added a paragraph for the limitations of our study in the Discussion to point on this (lines 639-651).

2. This research team previously demonstrated through animal experiments that the Japanese encephalitis virus (JEV) can undergo direct transmission among pigs via the oro-nanal route. However, in this manuscript, when conducting consecutive infections of JEV among pigs via direct transmission, they utilized the virus from the serum of previously infected pigs to infect the next batch of pigs (via the intranasal route), which could very likely introduce bias into the research results.

**Authors:** Serum was selected as the source of the virus for reasons of practicability, considering our limited possibilities to perform pig infection experiments at BSL3 level. From our previous work, we knew that only in the serum the virus is present in sufficiently high titers to have a good chance of continued infection of pigs during the passaging experiment. The use of oro-nasal fluids would have represented a considerable risk for lack of infection. To avoid introducing another bias, we opted against amplifying the virus by a cell culture amplification step. In addition, we hypothesized that the evolutionary pressure on JEV caused by single host passaging in pigs would be stronger that the pressure caused by different tissues in a pig (which has not been reported). Therefore, despite this limitation, our experimental layout should be able to pick up adaptations due to pig passaging. This was also supported by our sequencing data on swabs that were now added to the manuscript (see response to the specific points). To address this critique, we also added a comment in the Discussion to the limitation of the study (lines 639-651).

3. This manuscript only presented JEV in pig samples by quantifying viral RNA and did not present results regarding infectious virus. Particularly, the rapid rise of neutralizing antibodies in serum and the presence of substances in oro-nasal secretions that can degrade viral material suggest that measuring viral RNA alone may not exclude the influence of these factors.

**Authors:** The titration on C6/36 cells and MDMs were performed but for many samples we could not establish a titer as the samples were negative. We know from previous data that although JEV is present in the blood and pigs are efficient in transmitting the virus to mosquitos, the titers that are obtained in cell cultures are low and often under the detection limit. We agree that there are probably factors present in the serum and secretions that prevent infection in cell culture. These factors are present before the development of neutralizing antibodies and might for instance represent polyreactive immunoglobulins. We are now pointing on this problem in line 287-291.

4. When arboviruses infect vectors or vertebrate hosts, there is a phenomenon known as tissue-associated selection. However, in this manuscript, the analysis of the effects of direct transmission of JEV among pigs on the molecular level only focused on the virus in serum, which may not accurately reveal the actual situation.

**Authors:** Both serum and the swab samples were send to NGS. Although the coverage of the swabs was quite low and sometimes too low to analyze the viral sequences, this data does not point on a major tissue specific selection as the SNV frequencies in serum and swabs samples correlated well (new Table S6 and S7). We have added this supplementary data, text in lines 477-482 and a new paragraph in the Discussion (lines 639-651).

5. The authors also express doubts about the significance of direct transmission of JEV among pigs in field settings. In Figure 6 and their previously published paper, they demonstrate poor effectiveness of direct transmission. Additionally, some literature indicates that JEV viral RNA can be detected in oro-nanal secretions collected from pigs in the field, but infectious virus cannot be detected. Therefore, the attempt in this paper to conduct 10 passages of JEV transmission among pigs through direct (vector-free) transmission seems somewhat unrealistic.

**Authors:** Our previous data investigating direct transmission between pigs showed ranges of 20% or 100% of infectivity with the Nakayama strain in two independent experiments. We also showed previously the experiment oronasal infection does not required a high dose (Ricklin et al., 2016, Nat. Com.). Given the low density of pigs and high ventilation rate in our animal units, one could argue that this could be higher in field situation. With the very high number of pigs per pen that can be found in some of the pig production systems such as in China (sometimes over 100’000 animals in a production system) it is very difficult to estimate the risk of 10 passages in field situations. Also other diseases circulating in such a farm are likely to affect the transmission rate. Therefore, in our opinion, the data available was concerning enough to justify such investigations. Furthermore, virus adaptation might already occur after a few passages which could cause a continues increase in direct transmission rate. These aspects are discussed in lines 545-558.

6. This manuscript presents numerous research results, but most of the segments provide descriptions and explanations without integrating the results into a more comprehensive interpretation and discussion. This is indeed regrettable.

**Authors:** By replying to the specific comments, we hope to have improved the Discussion.

**Reviewer #3:**

Nothing

**Part III – Minor Issues: Editorial and Data Presentation Modifications**

**Reviewer #1:**

MATERIALS AND METHODS
Line 131-132: Please provide more details on how pig infection was conducted or provide references.

“Swabs and serum samples were taken at 0, 3 and 4 days post-infection (dpi)” – Please clarify when exactly day 0 swabs were taken, before or after infection, how deep swabs were taken, and what was done with swabs immediately after collection (frozen directly or diluted, if diluted then how, etc.).
“The day 4 serum was used for infection of three new pigs intranasally, keeping the lines separated (A, B and C).” – Please provide the volume of the sera for infection.
From the provided description, it is unclear what “lines” and “A, B, and C” are. Explanations are provided in the text below, but now it is confusing. Please change the language for clarity.

**Authors:** In reply to the above four points, we have improved this chapter to specifically address the missing information (lines 132-154).

Line 213: “P0 sample …” – it is not clear what is P0. Is it the JEV inoculum stock?
If P0 is JEV inoculum stock, please go through the entire text, figures, and figure legends, and change P0 to the “inoculum” or “initial JEV stock”, or something else because P0 in the given context is confusing and misleading.

**Authors:** We prefer to keep the term P0 because it refers to the counting of all passages making it clear that this is the starting inoculum. For clarification, we now indicate in each Figure Legend that P0 represents the initial JEV stock.

S8 Methods – more details are needed on how JEV isolated from serum was titrated for subsequent growth curves.

**Authors:** To clarify the different methodologies used in the in vitro assays, the viral titration is now separately explained in the S8 Methods. In addition, the immunoperoxidase staining for JEV infected cells, is now described in S9 Methods. The test in the corresponding chapters of the Materials and Methods was adapted to guide the reader. The different methodological assays, using one of these in vitro techniques are now referring to the Supplementary Methods 8, 9 and 10 (Supplementary Methods lines 132-159).

RESULTS

Line 242: Lines – A, B, C – should be shown on Fig 1a to easier refer text to the figure.

**Authors:** The lines were added in the schematic Figure 1a.

Line 247: “A statistical comparison of P0” – do you mean P1? There is no P0 in Fig 1a.

**Authors:** We removed the statistical analysis of the data shown in Fig 1, as we only have 2 data points for each condition and the ages of the pigs were highly variable during passaging. Therefore, the lines 268 to 282 were adapted.

Lines 254-256: “It is important to note that clinical differences may also have been influenced by age effects. The pigs used for the data shown in Fig 1 and 2 were from different litters. these reasons we further 257 characterized the viruses isolated from P1-P10.” – It is not clear what the authors mean here: “…. pigs used for the data shown in Fig 1 and 2,” does it mean that in addition to the JEV passage represented in Fig 1, additional animal experiments were conducted specifically for data conducted in Fig 2? If yes, these additional experiments should be described in MM. If not, please edit the text to clearly deliver information that Fig 1 and 2 represent data from the same animal experiment, and that pigs used in different passages (P1, P2, etc., Fig 1) have different ages.

**Authors:** No additional experiments were performed. The data in Fig 2 was created with virus stocks generated from the serum samples collected in the frame of the experiment described in Fig 1. The lines 287-292 were rewritten for clarification.

The age of pigs in each passage should be clearly described in MM.

**Authors:** The ages of all pigs for the two animal experiments can now be found in the S1 Table.

Fig 2 - the title, figure, and legend should clearly describe the sample type. It is unclear whether these are serum, nasal swab, or tissue samples.

**Authors:** The figure legend was adjusted accordingly.

Line 291: (Fig 3b-h) – “h” should be removed.

**Authors:** The line 333 was changed accordingly to Fig 3b-g.

Line 300: Fig 2h – change to Fig3h.

**Authors:** Line 342 was changed accordingly.

Line 359: Please edit the section title.

**Authors:** Line 486 was changed instead (first line of the paragraph).

Line 361: “In P0, 220 nucleotide deviations from the consensus Laos sequence were found.” – Here, the authors compare the inoculum stock sequences to the reference sequence available in NCBI (GenBank: KC196115.1); is it correct? Please clarify and edit the text for clarity.

**Authors:** Yes, the text was adjusted for clarification (lines 403-406).

Line 362: “During passaging, additional 8% of the nucleotide positions mutated.” – Here, NGS data from comparing in vivo JEV sequences as referenced to the de novo in-house generated inoculum JEV stock-specific sequences are described; is it correct? Please clarify and edit the text.

**Authors:** Yes, this is correct, and the text was adjusted for clarification (line 405-406).

**Reviewer #2:**

Specific comments:
1. Line 25 and 68: The role of birds in the transmission of Japanese encephalitis virus (JEV) remains unclear because current evidence mostly consists of indirect evidence, including antibody positivity rates and results from animal experiments. If birds do indeed serve as natural reservoirs, it raises the question of why there are not many JEV gene sequences isolated from birds in GenBank.

**Authors:** We have adapted the text (lines 68-70) in response to this comment.

2. Line 26: Currently, vector-free transmission of JEV has only been observed in experimental pigs and has not yet been confirmed in the field. It is suggested to rephrase it as "...observed in experimental pigs."

**Authors:** The text was changed as requested (see lines 26-27).

3. Line 132: "The day 4 serum was used for infection of three new pigs intranasally...". Explanation is needed as to why serum virus was used instead of virus from oro-nasal secretions and why the intranasal route was chosen over the oro-nasal route

**Authors:** We briefly explain the selection of serum in lines 144-145, and address this issue in the Discussion as mentioned above. Furthermore, the explanation for the switch to the intra-nasal route was added in the lines 146-151.

Additionally, the inoculation dose (ul or ml/animal) should be specified. Here, when referring to "serum," it should be clarified how it was prepared after blood collection. Was it allowed to clot, or was an anticoagulant added? Typically, the serum is obtained by centrifugation after blood clotting, but the clotting process can significantly reduce the amount of infectious virus, making it less suitable for samples with low viremia, including JEV.

**Authors:** The inoculum volume can be found lines 144. The serum preparation was further specified in the lines 141-145.

4. Line 184 and Fig 2: It should be explained why both mosquito C6/36 cells and pig PEDSV.15 cells were used for JEV virus titration.

**Authors:** The usage of both cell types is explained in the lines 207-210 as well as in lines 301-302.

5. Line 190: The S7 methods section does not describe the TCID50 measurement method but rather the Focus Forming Units (FFU) measurement method. Throughout the manuscript, the quantification of infectious virus is stated in terms of TCID50/ml. Please clarify which method is used. Typically, C6/36 cells do not die after JEV infection. How was the TCID50 quantification performed?

**Authors:** For clarification of the titration methods, the FFU is described in the section S7 methods, the viral titration (TCID50 measurement) is newly described in the S8 methods and the immune-peroxidase staining (used in both (S7 and S8) methods) is now described in the S9 methods. The text was adapted to provide this information to the reader.

6. Lines 197-198: This manuscript only specifies that viral RNA used for viral genome analyses is derived from serum or swab samples. Figure 7 does not specify the source of viral RNA, while Figures 8 and 9 indicate that viral RNA is from serum. The source of viral RNA could significantly impact the obtained results, so it should be clearly stated.

**Authors:** We have added the requested information to the legend of Figure 7 (it is from serum). In the revised manuscript we have in addition added the data on SNV available for the swab samples (new Table S6 and S7).

If analyzing viral RNA from a single tissue or organ source, the reasons for this choice should be explained, along with a discussion of potential limitations.

**Authors:** As requested, we have discussed these issues in lines 639-651.

7. Line 250 and others: Regarding the units for viral RNA quantification, throughout the manuscript and figures, it is stated as viral copies/μl serum, with values reaching up to ~105, indicating up to ~108 per ml. This value is remarkably high for samples from JEV-infected pigs, so please confirm if this is accurate. In their 2016 article published in Nature Communications, the authors used relative quantitative RT-qPCR, defining 1 TCID50 as 1 RNA unit. In this manuscript, absolute quantitative RT-qPCR is used. Could authors provide relevant information to facilitate the estimation of the amount of infectious virus?

**Authors:** We can confirm these numbers. 10^4^ viral copies/μl serum correspond to 2x10^4^ U/ml (1 TCID50 as 1 RNA unit). This information was added to the manuscript in line 175-176 and lines 272-274.

8. Line 255: The authors mention that the clinical presentation of pigs may be influenced by their age; however, experimental pig ages are not provided in the text, especially concerning the ages of pigs in different passages.

**Authors:** The age of each pig at the day of infection is now listed in the S1 Table.
9. Lines 273-279: It should be explained why all serum viruses need to be rescued on macrophages before conducting the replication capacity comparison. Additionally, the impact of this additional step on in vitro experiments should be discussed and addressed. Utilizing infectious virus directly from serum, rather than the rescued virus, for in vitro experiments would more accurately reflect real-world conditions. The results obtained from in vitro experiments are not compared and discussed alongside in vivo results and viral genome analysis results.

**Authors:** This is now addressed in the lines 288-293. Unfortunately, it was impossible to use the serum directly for the cell culture tests due to low titres. The reason why we did not further discuss the in vitro results with the genome analysis was that we found no meaningful associations.

10. Lines 139 and 283: In the in vivo experiment presented in Fig 3, P10B and P10C were mixed in a 1:1 ratio. However, the in vitro results shown in Fig 2 indicate that the viruses from line B and line C are different. It may not be suitable to mix them for subsequent experiments. The experimental challenges and potential impacts on the results should be explained and discussed.

**Authors:** The mixing was performed with the rational to reduce the number of animals required (which is limited due to the BSL3 conditions). In our opinion this mixing is unlikely to affect the outcome of the animal experiment. If for instance line P10B would contain variants with increased fitness these would quickly dominate and outgrow variants with lower fitness. The SNV data shown in Fig 9 demonstrates that it is possible to track such variant selection. Therefore, mixing the two lines generates valuable additional information by assessing the direct competition between the viral variants. This is explained in the results (lines 462-476) and taken up in various parts of the Discussion when selected haplotypes and SNV are discussed.

11. Line 312: Analysis of cytokine profiles revealed that JEV infection of pigs by intranasal route induces a Th1 response, but this finding is not compared, explained, or discussed alongside the subsequent Gene Set Enrichment Analysis (GSEA) results. Furthermore, there is no discussion comparing these results with those from other JEV animal models, infection routes, or analyses of clinical samples from humans.

**Authors:** We are now extensively discussing the immune responses data (lines 516-539).

12. Lines 315-317 and Fig 4i: This experiment found that regardless of the experimental group, the time and intensity of neutralizing antibody responses were the same. However, Fig 3C shows differences in clinical presentation. How can this be explained? What role might cytokines or T cell responses play?

**Authors:** This is discussed in the lines 516-523.

13. Line 352 and Fig 6: The experimental results indicate that direct transmission via the oro-nasal route is less effective. The authors attribute this to the density of housing, but cannot rule out the possibility that it is due to the lower quantity of infectious virus present in oro-nasal secretions. In Fig 1D and 1E, it is speculated that the quantity of infectious virus in serum is much higher than in oro-nasal secretions. Therefore, using serum virus for 10 passages of direct transmission may significantly affect the bottleneck effect, thereby rendering the results of this manuscript unable to reflect reality (as mentioned in general comment 2). While the results in Fig 6 show no difference in direct transmission between P1* and P11 viruses, the results in Fig 3E indicate differences in nasal shedding. How can this be explained?

**Authors:** Only Fig. 3e should be used to interpret the possible impact of oronasal shedding on transmission because this data set contained sufficient animals that all had the same age. In addition, these were the animals which were co-housed with the sentinel pigs. The data indicates adaptations towards extended shedding from day 8 on, but not towards increased shedding in the first week post infection. In addition, the P11 group showed stronger symptoms than the P1* group, which is expected to reduce the contacts to the sentinel pigs. While we cannot exclude bottleneck effects for the virus to replicate in tissues of the oro-nasal cavities, we have no evidence for this from our sequence data on the swab samples that are now described in the manuscript. This is described in lines 477-482 discussed in lines 647-651.

14. Line 359 and Fig 7: The authors did not provide basic information regarding the NGS analysis, such as the number of reads, the proportion of reads belonging to JEV, and the resulting coverage depth across the viral genome.

**Authors:** The information is now provided in the S2 Table and referred to in lines 231-233.

Why were reads filtered for a frequency above 35%?

**Authors:** We were interested in SNV that were selected during pig passages which would favour a higher threshold. Nevertheless, when looking at the data we also found some SNV that reached frequencies around 40-60% but then declined again along the passages. In addition, a few SNV emerged late during passages that we also wanted to visualize. The 35% cutoff was selected to visualize those events and at the same time avoid showing too many plots. We refer to the supplementary data for readers interested in SNV that never reached that threshold. We have added this information to the text lines 410-413.

The results obtained from Fig 7b and 7c should be integrated, explained, and discussed alongside the results from Fig 1, 2, and 3.

**Authors:** We find it too speculative to discuss in more detail the data shown in Fig. 7b and c with respect to the data in Figs 1-3 given that it deals with just an overview and introduction to the sequence data. Nevertheless, there is a paragraph on this in the Discussion (lines 571-577). In addition, the data is explained and discussed in context of variant/SNV selection (lines 580-582).

15. Line 415 and Fig 9: P10B and P10C were mixed at a ratio of 1:1 based on the infectious virus (line 139: forming 1.8X105 TCID50), which does not necessarily reflect a 1:1 ratio at the viral genome level. The authors should explain and discuss the potential impact of this discrepancy.

**Authors:** As explained above the mixing of 10B and 10C had the aim to identify SNV/haplotypes with a selective advantage. A 1:1 ratio was selected as the most logical approach. We are aware that it does not mean a 1:1 ratio at the genomic level but this was impossible to achieve because of the quasispecies nature of these passages. For the calculations the mutational frequencies in the original P10 stocks were divided by two because of the volume doubling. So, if a mutation in P10B was present at 40% in 10B and 0% in 10C, its frequency was calculated as 20%. This was then compared to the frequencies in the P11. So, if the frequency was below 20% we concluded that there was no selection. We have adapted the text to clarify this (lines 466-468).

16. Comparative analysis of results from Fig 1 and 3 requires explanation and discussion, highlighting three points:

(1) Why is the viral RNA quantity in Fig 3E and 3F significantly higher than in Fig 1E?

(2) The viral RNA quantity in Fig 3D, 3E, and 3F is similar, but why is it noticeably higher in Fig 1D than in Fig 1E?

(3) While the overall trend in Fig 1C is downward, why is P11 higher than P1* in Fig 3C?

**Authors:** Figure 1 is not suited to conclude on possible viral phenotype changes of over time. To avoid this impression, we have removed the statistical analysis and now made this clear in the text (lines 282-283). There are four main reasons for this. First, with each passage the pigs were older, and this can influence the results, in particular the clinical scores. Second apart from the very first infection, the infectious doses were not controlled. Third, we only have data for the first four days of infection which is not sufficient to conclude on the parameters assessed. Forth, for P5-P6 in Figure 1 we only have two animals which make this comparison even more difficult given the variation between animals.

With respect to the viral RNA in the swab it should also be noted that they peak around day 5-6, but if one focusses on day 3 and 4 the differences are not remarkable. In addition, Figure 1E represent oronasal swabs whereas in Figure 3e/f either nasal or oral swabs. The average of all the viral RNA amounts at day 3 and day 4 for Figure 1E is 810 viral copies/µl, for Figure 3E 3466 viral copies/µl and for Figure 3F 721 viral copies/µl.

**Reviewer 3:**

specific comments

**Introduction**

Line 61: give reference for mortality rate.

**Authors:** They were combined with the neuropsychiatric sequelae one line below; however we added them now as well in line 61.

Line 63: vertebrate ‘species’. I would not refer to transmission as dual-host – it is host-vector, also drop ‘alternating’ as you say cycling – same thing.

**Authors:** The text was exchanged as requested (lines 63-64)

Line 67: must be adapted to both host and vector (refer to mosquitoes as vectors, not hosts).

**Authors:** The text was exchanged as requested in line 68.

Line 69: ‘and produce an immune response’.

**Authors:** This was added to line 69-70.

Line 76: just ‘direct transmission’.

**Authors:** This was corrected in line 77.

Line 77: omit ‘worrisome’.

**Authors:** This was deleted form the text (line 78).

Lines 80 to 82: Not necessarily true – this depends on pig population turnover and seasonal dynamics of JEV. In some regions if there is only seasonal transmission season, this could result in a build-up of susceptible animals between outbreaks. I would say it is less likely in many endemic areas because pig farming is not intensive.

**Authors:** The text was adjusted (lines 81-83).

*Methods*

Statistical analysis: it would be good to elaborate on the statistics in more detail. Clearly outline each hypothesis/ question each test is addressing. For example, ‘the analyses related to the *in vivo* passaging…’ separate this out into each analysis – what were the dependent variables?

**Authors:** We included information about the statistical tests in each Figure legend to directly link the figure with the statistical analysis.

From reading the results it looks like analysis was done separately for individual time points for the dependent variables in Fig. 1, Fig. 2 and Fig. 3. Or, for the data in Fig. 1 did you only compare values at day 3-4? Ideally, you should treat time as a continuous variable and account for repeated measures in your statistical analyses. How were the curves generated in Fig. 3?

**Authors:** We removed the statistics from Figure 1, as two datapoints make it questionable for statistical analysis. This is also difficult due to additional uncontrolled variables such as increasing age of the animals and uncontrolled infection dose. This data set does not allow any conclusion on changes over the passages.

For Figure 2 and 3 we used statistical significances at individual days post infections to directly compare these time points. The data in Figure 2 points on two major effects. Differences at early times points (in particular at 24h) in C6/36 and growth to higher titres in PEDSV.15 (visible at 48 and 72h). For additional stats relating to Figure 2 see below.

For Figure 3 we also included an analysis over the whole time of the experiment with the area under the curve (significance indicated in the figure legend). The area under the curves were then compared by an unpaired t test with Mean, SEM and N. The area is therefore used as mean, the standard deviation as SEM and the N is indicating the degrees of freedom.

In response to the suggestion of the reviewer, we also performed a repeated measure Anova with Geisser-Greenhouse correction. The results correspond with the significances obtained by the area under the curve analyses, with exception for the temperature data that is now also significantly different between the groups. The results text indicates the two tested methods now.

For *in vitro* growth curve analysis for example, you could follow the approach in Wang and Bushman (2006) A statistical method for comparing viral growth curves. Journal of Virological Methods. 135, 118-123. However, note this approach only examines the exponential phase of growth curves.

**Authors:** The exponential phase of the growth curve does not start for all of our growth curves at the same point. Therefore, we performed a linear regression and compared the different slopes by Dunnett’s multiple comparison test. The Result text was adapted lines 312-322 and the statistical data is given in S3 Table.

*Results*

Linked with the comments on statistics, it would be good to focus in the description of the results on effect sizes and present more quantitative findings. For example, lines 269 – 270 – what was the observed difference in growth rate? Line 291: significantly increased body temperature – but what was the difference in body temperature?

**Authors:** In our opinion, adding such numbers to the text makes it difficult to read and we prefer that the reader takes this information from the figures, which also offers the advantage of the visual representation of the variation. We hope this is acceptable for the Reviewer and the Editor.
