# Supplementary material for: Fitness adaptations of Japanese encephalitis virus in pigs following vector-free serial passaging
Source: PLoS Pathog. 2024 Aug 26;20(8):e1012059. doi: 10.1371/journal.ppat.1012059 (PMC11379391; doi:10.1371/journal.ppat.1012059)
Supplement: Attachment — Submitted filename: Point by Point Reply to the Reviewers.docx [file ppat.1012059.s016.docx]

**Point by Point Reply to the Reviewer's Responses to Questions**

Authors: We are grateful to the Editors and Reviewers to further consider our manuscripts for publication in PLOS PATHOGENS. Please find our point-by-point reply the the Reviewer’s comments.

**Part I - Summary**
Please use this section to discuss strengths/weaknesses of study, novelty/significance, general execution and scholarship.

Reviewer #2: The revised manuscript has undergone significant improvement. Despite the authors' inability to enhance the experimental design or conduct further experiments due to practical constraints, they have included additional explanations in the discussion concerning potential limitations that could affect the study. Therefore, while the authors' response is less than ideal, it is deemed acceptable.

1. Although conducting experiments involving infection between the vector and pigs was impractical for logistical reasons, conducting virus infection experiments solely in the vector (which is comparatively simpler) would likely enhance the interpretation of the research findings.
2. Regarding concerns about potential bias arising from using virus extracted from the serum of previously infected pigs to infect the subsequent batch of pigs, the authors were fortunate that the NGS results from serum and swab samples in this study were similar (Table S6 and S7), thus partially addressing the aforementioned concerns.

Authors: Experiments regarding vector competence for the passages viruses are in planing and we have identified a collaboration partner that has the required expertise. Nevertheless, It will not be possible to add this data to the manuscript as we anticiplate that it will take at least six month to be generated. Nevertheless, the data will be available for the reader in the near future and will refer to this manuscript.

Reviewer #3: As per previous review

**Part II – Major Issues: Key Experiments Required for Acceptance**
Please use this section to detail the key new experiments or modifications of existing experiments that should be absolutely required to validate study conclusions.

Generally, there should be no more than 3 such required experiments or major modifications for a "Major Revision" recommendation. If more than 3 experiments are necessary to validate the study conclusions, then you are encouraged to recommend "Reject".

Reviewer #2: No further comment.

Reviewer #3: NA

**Part III – Minor Issues: Editorial and Data Presentation Modifications**
Please use this section for editorial suggestions as well as relatively minor modifications of existing data that would enhance clarity.

Reviewer #2: No further comment.

Reviewer #3: Introduction
In the previous comments I requested to refer to mosquitoes as vectors not hosts to be clear what you are referring to, but this remains the case on line 64 and on line 66. This is confusing because JEV can circulate in multiple host species. Please be clear in the text you mean host and vectors not two hosts.
Paragraph on Line 80: You need to better explain why DT would be less likely than mosquito-host transmission when there are more resistant individuals in the population compared to a fully susceptible population. In addition, line 80 specifically is poorly phrased – you talk about DTs being rare in endemic areas but then give evidence for this based on piglets in Cambodia. It would be better to start this section by explaining the importance of resistance generally – then giving examples of where resistance comes from (maternal antibodies, vaccine, exposure).

Authors: We have changed the text regarding vector/host in lines 64 and 67/68 as requested. We have also reworded the text describing why the rate of DT should be impacted by pre-existing immunity in pigs (see lines 75-81).

Methods
In the previous review, I requested more detail as to the statistics carried out in the methods, but there now seems to be even less! I recommend fully writing out the statistical analyses for each hypothesis to be tested within the methods. This helps the reader to follow what you have done and why. This is particularly important in a study such as this which has a lot of components. I understand that you have put the stats in the figure legends - but you wouldn't think to do that for other parts of the methods, so why the statistical analysis methodology?

Authors: All statistical tests are now described in the Materials and Methods section as requested.

Results
Please omit the use of ‘interestingly’ and ‘surprisingly’ in the results
There is some repetition on lines 286 and 299

Authors: We have remove these words from the Results section as requested.
